# Lack of association between stavudine exposure and lipoatrophy, dysglycaemia, hyperlactataemia and hypertriglyceridaemia: a prospective cross sectional study

**DOI:** 10.1186/1742-6405-7-23

**Published:** 2010-07-14

**Authors:** Phumla Z Sinxadi, Jan-Stefan van der Walt, Helen M McIlleron, Motasim Badri, Peter J Smith, Joel A Dave, Naomi S Levitt, Gary Maartens

**Affiliations:** 1 Department of Medicine, Division of Clinical Pharmacology, University of Cape Town, K45 Old Main Building, Groote Schuur Hospital, Observatory, 7925, Cape Town, South Africa; 2 Department of Medicine, Clinical Research Support Unit, University of Cape Town. J45-46 Old Main Building, Groote Schuur Hospital, Observatory, 7925, Cape Town, South Africa; 3 Department of Medicine, Division of Diabetic Medicine and Endocrinology, University of Cape Town. J47 Old Main Building, Groote Schuur Hospital, Observatory, 7925, Cape Town, South Africa

## Abstract

**Background:**

Stavudine continues to be widely used in resource poor settings despite its toxicity. Our objective was to determine association between plasma stavudine concentrations and lipoatrophy, concentrations of glucose, lactate and triglycerides.

**Methods:**

Participants were enrolled in a cross-sectional study with lipoatrophy assessment, oral glucose tolerance test, fasting triglycerides, finger prick lactate, and stavudine concentrations. Individual predictions of the area under the concentration curve (AUC) were obtained using a population pharmacokinetic approach. Logistic regression models were fitted to assess the association between stavudine geometric mean ratio > 1 and impaired fasting glucose, impaired glucose tolerance, hyperlactataemia, hypertriglyceridaemia, and lipoatrophy.

**Results:**

There were 47 study participants with a median age of 34 years and 83% were women. The median body mass index and waist:hip ratio was 24.5 kg/m^2 ^and 0.85 respectively. The median duration on stavudine treatment was 14.5 months. The prevalence of lipoatrophy, impaired fasting glucose, impaired glucose tolerance, hyperlactataemia, and hypertriglyceridaemia were 34%, 19%, 4%, 32%, and 23% respectively. Estimated median (interquartile range) stavudine AUC was 2191 (1957 to 2712) ng*h/mL. Twenty two participants had stavudine geometric mean ratio >1. Univariate logistic regression analysis showed no association between stavudine geometric mean ratio >1 and impaired fasting glucose (odds ratio (OR) 2.00, 95% CI 0.44 to 9.19), impaired glucose tolerance (OR 1.14, 95% CI 0.07 to 19.42), hyperlactataemia (OR 2.19, 95%CI 0.63 to 7.66), hypertriglyceridaemia (OR 1.75, 95%CI 0.44 to 7.04), and lipoatrophy (OR 0.83, 95% CI 0.25 to 2.79).

**Conclusions:**

There was a high prevalence of metabolic complications of stavudine, but these were not associated with plasma stavudine concentrations. Until there is universal access to safer antiretroviral drugs, there is a need for further studies examining the pathogenesis of stavudine-associated toxicities.

## Introduction

Stavudine is no longer recommended as part of first line combination antiretroviral therapy (ART) because of a high cumulative risk of toxicity, notably symptomatic hyperlactataemia/lactic acidosis, lipoatrophy, and peripheral neuropathy [1,2]. In addition, stavudine causes dyslipidaemia and insulin resistance, and is an independent risk factor for the development of new onset diabetes mellitus [3]. Although the World Health Organization (WHO) ART guidelines for resource-limited settings urge countries "to begin planning to move away from stavudine-containing regimens" [4], stavudine continues to be widely used in standardised first-line regimens in low- and middle-income countries as it has a low acquisition cost, is available in fixed dose combination formulations and does not require laboratory monitoring for toxicity.

In 2006, WHO recommended reduced doses of stavudine following the findings of a systematic review that lower doses caused less toxicity without reducing efficacy [1,5]. Most, if not all, of stavudine's adverse drug reactions are thought to be mediated by mitochondrial toxicity and to be dose related [6,7]. Therefore it is reasonable to assume that higher plasma concentrations of stavudine might be associated with more toxicity. However, there are "no clear plasma concentration-effect relationships" with nucleoside reverse transcriptase inhibitors like stavudine, which are pro-drugs that require intracellular tri-phosphorylation for antiviral activity [8]. A retrospective study from the Netherlands reported a correlation between lipoatrophy and higher stavudine plasma concentrations [9], but data correlating stavudine plasma concentrations with other metabolic adverse drug reactions are lacking.

We investigated whether there was an association between stavudine plasma concentrations and lipoatrophy or concentrations of glucose, lactate and triglyceride in a population where stavudine use is likely to be widespread in the medium term: African HIV-infected adults.

## Methods

### Study design and participants

We conducted a prospective cross sectional study between February 2007 and January 2008. Ambulatory HIV-infected African black adults who presented for a routine follow up visit at public sector antiretroviral clinics in Cape Town were recruited by convenient sampling. Participants were eligible if they were on stavudine-based therapy for a minimum of 6 months. Participants with renal or hepatic disease, active opportunistic infections, known diabetes or dyslipidaemia, or self-reported non-adherence were excluded. All participants gave informed consent. The University of Cape Town research ethics committee approved the study.

### Clinical and laboratory evaluations

Participants fasted overnight and underwent an oral glucose tolerance test (OGTT). Impaired fasting glucose (IFG), impaired glucose tolerance (IGT) and diabetes were defined according to the American Diabetes Association criteria [10]. Fasting triglycerides were determined at 0 min of the OGTT. Hypertriglyceridaemia was defined according to the NCEP III criteria [11]. Finger prick lactate was measured before the glucose loading using the Accutrend^® ^lactate meter (Roche, Basel, Switzerland). Hyperlactataemia was defined as a lactate concentration greater or equal to 2.5 mmol/L.

Lipoatrophy was determined by self-reported peripheral fat loss using a validated questionnaire [12]. Lipoatrophy was rated as absent (score = 0), mild (noticeable on close inspection, score = 1), moderate (readily noticeable by participant, score = 2) or severe (readily noticeable to a casual observer, score = 3) in each of four areas (face, arms, legs and buttocks). The lipoatrophy score could range from 0 to 12. Lipoatrophy was regarded to be present if the score was 1 or above.

Self reported adherence was determined using a standard 4-day adherence questionnaire administered by trained field workers [13]. We reviewed medical records to determine duration on antiretroviral therapy and current CD4+ lymphocyte counts and viral load. Current CD4+ count was regarded as the count measured within 3 months of the study visit.

We measured plasma stavudine concentrations at 0, 30, and 120 minutes of the OGTT. We collected the blood samples using heparinised tubes that were immediately placed on ice until centrifugation within 4 hours, and then kept in a minus 80°C freezer until analysis. Stavudine was assayed by liquid chromatography tandem mass spectrometry using a validated method on an API 4000 mass spectrometer. The mobile phase consisted of gradient of acetonitrile and 0.5% glacial acetic acid. Chromatography was performed on a Phenomonex Synergi fusion C18 column maintained at 25°C. Reserpine was used as an internal standard. 50 μL of each sample was precipitated with acetonitrile containing the internal standard, centrifuged and 5 μL of the supernatant injected onto the column. Standard curves in the range 0.02 - 6 μg/mL and appropriate quality control samples were run with each batch. The lower limit of quantification was 20 ng/mL. Inter- and intra-day coefficients of variation were below 9% for all quality control samples.

### Pharmacokinetic analysis

The aim of the pharmacokinetic analysis was to obtain a prediction of each participant's apparent stavudine clearance (CL/F) for calculation of the area under the concentration curve (AUC), where AUC = dose (ng)/clearance (L/h). The data were analysed using nonlinear mixed effects modelling with NONMEM^® ^(version VI level 2.0; ICON Development Solutions, Ellicott City, MD, USA). Given the sparse data (1-3 observations per participant), a model developed using rich stavudine concentration data from a separate study our group has conducted of African adults from the same community was used [14]. The population pharmacokinetic parameter estimates were fixed to: apparent clearance 17.8 L/h/70 kg^3/4^ (between subject variability 17%CV), apparent volume of distribution 33.5 L/kg, first-order absorption rate constant 11.1/h (between subject variability 125%CV), absorption lag time 0.41 h, proportional residual variability 27% and additive residual variability 10 ng/mL. Individual pharmacokinetic parameter sets were then obtained using Bayesian estimation given these model parameters and the observed data. The geometric mean ratio (GMR) was calculated by comparing the individual log-transformed AUC to the mean log-transformed AUC of the overall population.

### Statistical analysis

Means (standard deviation (S.D)) and medians (interquartile range) were used to describe parametric data and non-parametric data, respectively. Categorical data were compared using χ^2 ^test (or Fisher's exact test), and continuous data were compared using student's T-test or Mann-Whitney test, whichever was appropriate. Logistic regression models were fitted to assess the association between GMR > 1 and IFG, IGT, hyperlactataemia, hypertriglyceridaemia and lipoatrophy. Linear regression models were fitted to assess the association between log-transformed stavudine area under the curve and the following variables: concentrations of glucose, lactate and triglycerides, and lipoatrophy scores. All tests were two-sided, and a P-value < 0.05 was considered significant. Analyses were performed using SPSS (version 17, SPSS Inc, Chicago, Illinois, USA)

## Results

Forty seven black participants were included for the analysis. Median (IQR) age was 34 (30-38) years. Thirty nine participants were female. Median (IQR) weight and body mass index were, 61.0 (54.4 to 73.8) kg and 24.5 (21.5 to 30.4) kg/m^2^, respectively. Median waist to hip ratio was 0.85 (0.80 to 0.92). Median (IQR) current CD4 count was 304 (234-516) cells/μL. Twelve participants were virologically suppressed, 6 had viral load above 50 copies/mL and 29 had no viral load data. Forty and seven participants were on 30 mg and 40 mg of stavudine, respectively. Twenty six, twenty and one participants were on efavirenz, nevirapine and lopinavir, respectively. All participants were on lamivudine. The median (interquartile range (IQR)) fasting glucose concentration was 4.9 (4.7 to 5.4) mmol/L and the mean (standard deviation (sd)) 2 hour glucose concentration was 5.34 (1.43) mmol/L. Nine and two participants had IFG and IGT, respectively. The mean (sd) lactate concentration was 2.26 (0.78) mmol/L and 15 participants had hyperlactataemia. The median (IQR) triglyceride concentration was 1.17 (0.85 to 1.60) mmol/L and 11 participants had hypertriglyceridaemia. The median (IQR) lipoatrophy score was 0 (0 to 9) and 16 patients had lipoatrophy.

**Figure 1:**
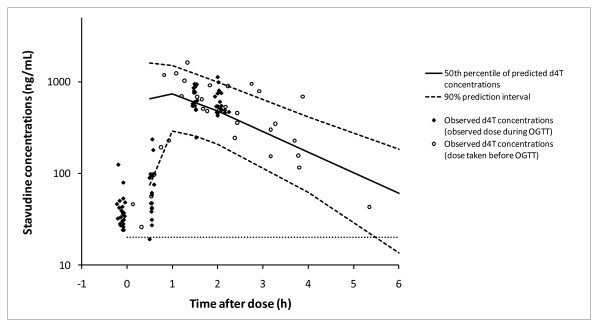
**Plasma stavudine concentrations vs time after dose, collected during OGTT in 47 HIV-1 infected participants**. OGTT = oral glucose tolerance test, d4T = stavudine. The solid line indicates the median predicted concentrations and the dashed lines the 90% prediction interval of a model developed using rich stavudine concentration-time data. The lower limit of quantification (20 ng/mL) is shown by a dotted line. Open circles indicate stavudine concentrations when stavudine was taken before the OGTT, and the solid diamonds are stavudine concentrations collected when the stavudine dose was taken during the OGTT (i.e. the 0-hour OGTT was collected pre-dose).

A total of 122 stavudine concentrations from 47 participants were analysed. Eleven participants had no pre-dose concentrations because they took their stavudine morning doses prior to the OGTT. The observed stavudine concentrations plotted against the model predictions are shown in Figure 1. Stavudine exposure was expressed with the calculated AUC. The median (interquartile range) stavudine AUC was 2191 (1957 to 2712) ng*h/mL. The mean (standard deviation) log-transformed AUC was 3.36 ± 0.10 ng*h/mL. 22 participants had a GMR greater than 1.

We found no association between log-transformed stavudine AUC and metabolic parameters expressed as continuous variables (Table 1). We also showed no association between stavudine geometric mean ratio >1 and abnormal metabolic parameters expressed as categorical variables (Table 2). We found an association between duration and triglycerides concentrations (beta coefficient 95%CI = 0.02 (0.01 to 0.04) p = 0.004. No significant association was found between duration and glucose and lactate concentrations as well as lipoatrophy scores.

**Table 1:** Univariate linear regression analysis of stavudine log-transformed AUC and metabolic parameters

Variable	Beta coefficient (95% CI)	p-value
Fasting glucose	-0.02 (-0.06 to 0.04)	0.54
2 hour glucose	-0.02 (-0.04 to 0.00)	0.09
Lactate	0.00 (-0.04 to 0.04)	0.91
Triglycerides	0.00 (-0.05 to 0.05)	1.00
Lipoatrophy score	-0.00 (-0.01 to 0.01)	0.37

**Table 2:** Univariate logistic regression analysis of stavudine geometric mean ratio >1 and metabolic parameters

Variable	Odds ratio (95%CI)	p-value
Impaired fasting glucose	2.00 (0.44 to 9.19)	0.37
Impaired glucose tolerance	1.14 (0.07 to 19.42)	0.93
Hyperlactataemia	2.19 (0.63 to 7.66)	0.22
Hypertriglyceridaemia	1.75 (0.44 to 7.04)	0.43
Lipoatrophy score >0	0.83 (0.25 to 2.79)	0.83

## Discussion

Despite guidelines recommending that the use of stavudine be avoided because of its toxicity, it continues to play a critical role in scaling up antiretroviral therapy in resource poor settings. Therefore, studies examining pathogenesis of stavudine toxicity are still relevant. We found no association between stavudine AUC and the lipoatrophy scores or concentrations of glucose, lactate and triglycerides. To our knowledge this is the first study to evaluate the association between plasma stavudine concentrations and serum glucose, lactate and triglycerides. We found high prevalence of metabolic abnormalities in this black African cohort with a median duration of stavudine exposure of 14.5 months: lipoatrophy (34%), dysglycaemia (23%), hyperlactataemia (32%), and hypertriglyceridaemia (23%). We found an association between duration and triglycerides concentrations.

A meta-analysis from randomised control trials and cohort studies showed that switching from higher to lower doses of stavudine, or starting at lower doses, is associated with improvement in stavudine toxicity without loss of efficacy [5]. Switching to lower doses of stavudine was associated with decreased drug exposure, mitochondrial DNA repletion, partial reversal of lipoatrophy, improvement in lactate and lipids [5,15,16]. Therefore, stavudine toxicity is dose related. The lack of an association between stavudine AUC and all the metabolic abnormalities that we found can be explained as follows: First, like all nucleoside/nucleotide reverse transcriptase inhibitors (NRTIs), stavudine is a pro-drug that must be converted intracellularly into its triphosphate moiety (d4T-TP) to exert antiviral activity by competing with endogenous nucleotides to terminate HIV replication [17]. The d4T-TP also inhibits mitochondrial DNA polymerase gamma in a dose dependent manner in cells of various tissues, and effectively terminates mitochondrial replication with subsequent mitochondrial damage or depletion: the common pathway for stavudine related toxicity. Intracellular triphosphate concentrations, but not NRTI parent drugs, have generally been shown to have a good correlation with antiviral activity [17-21], as well as toxicity [22]. The cellular processes that affect the relationship between plasma NRTI and intracellular triphosphate concentrations include variation in expression of drug transporters, rate limiting steps or saturated phosphorylation steps, cell activation state, and drug interactions [17,19]. Second, clinical manifestations of stavudine toxicity are also influenced by host susceptibility such as age, sex, advanced HIV disease and genetic susceptibility [17,23-26]. Third, it seems that stavudine toxicity is cumulative, as it is shown to be dose related and is associated with prolonged duration on treatment [5,9,15,16]. Therefore, differences in plasma concentrations, if they exist, are likely to be small, and a very large study will be needed to detect the difference.

Although stavudine related toxicity is well documented, to date, few studies have investigated pharmacokinetic relationship with stavudine toxicity. Our findings are different to a case-control study conducted by ter Hofstede et al, which reported that cases with lipoatrophy had higher stavudine exposure than controls [9]. However, there were no statistically significant differences in geometric means of concentration ratios between the cases and controls. The discrepancy between their findings and ours could possibly be explained by differences in study design and participant selection. Ter Hofstede et al conducted a retrospective study. Exposure was represented by a time-adjusted concentration ratios derived from a historic population. In contrast, our study was conducted prospectively and we used stavudine AUC derived from individual clearances obtained from a pharmacokinetic model of intensively sampled participants from the same community [14]. The estimated AUC of 2191 ng*h/mL in our population is similar to that found in the richly sampled South African population we used for the population model [14] and to control patients from the US [27], but is higher than reported in Indian patients [28] or the Summary of Product Characteristics of Zerit [29]. It is possible that stavudine exposure is high in our population, which may account for the high prevalence of metabolic abnormalities we observed. However, the prevalence of metabolic abnormalities on stavudine-containing regimens that we found were comparable to other published studies with variable duration of follow-up and different additional antiretroviral drugs: lipoatrophy (20-42%) [30-33], dysglycaemia (3-25%) [34], hyperlactataemia (15-35%) [35], and hypertriglyceridaemia (22-71%) [36,37]. We found an association between duration and triglycerides concentrations and this has been reported before [36].

Our study had a few limitations. First, we measured stavudine concentrations in plasma and not the active intracellular triphosphorylated metabolite. Second, we used sparse sampling instead of intensive sampling. However, a population approach allowed us to predict individual AUCs, an acceptable measure of drug exposure. Third, we did not have data on genetic polymorphisms. Fourth, sample size of this study was small, and therefore might have insufficient power to detect relatively small effects of plasma concentrations on metabolic abnormalities. However, this sample size is larger than in other pharmacokinetic studies that have examined the association between stavudine concentrations and metabolic toxicity [9,22].

Future studies examining the pathogenesis of stavudine-associated toxicities should have adequate power and preferably be longitudinal. Relevant genetic studies should also be done in the populations where stavudine will still be used in the medium term. Physiologically based pharmacokinetic models that take into account the temporal fluctuations and intracellular cascade steps of plasma NRTIs and metabolites should be used to establish pharmacokinetic-pharmacodynamic relationships.

In conclusion, we did not find an association between stavudine exposure and metabolic complications. Despite guidelines recommending that the use of stavudine be avoided because of its toxicity, it is still widely used in resource poor settings. Until there is universal access to safer drugs, there is a need for further studies examining the pathogenesis of stavudine-associated toxicities.

## Competing interests

The authors declare that they have no competing interests.

## Authors' contributions

PZS participated in the study design, acquisition of data, data analysis and interpretation, and drafted the manuscript. JSvdW participated in study design, population pharmacokinetic analysis and helped to draft and critically revise manuscript. HMM participated in study design, data interpretation, and critical revision of the manuscript. MB performed statistical analysis and helped to draft and revise manuscript. PJS performed analysis of the samples and helped to draft the manuscript. JAD participated in study design, acquisition of data and critically revised the manuscript. NSL participated in study design and acquisition of data. GM conceived of the study, participated in study design, data interpretation, and critically revised manuscript. All authors read and approved the final manuscript.
